# Towards multi-criteria optimization of transducer configurations

**DOI:** 10.1186/2050-5736-3-S1-P46

**Published:** 2015-06-30

**Authors:** Dinesh Acharya, Tobias Preusser, Joachim Georgii

**Affiliations:** 1 Fraunhofer MEVIS, Bremen, Germany; 2 Fraunhofer MEVIS/Jacobs University Bremen, Bremen, Germany

## Background/introduction

One of the key challenges of using Focused Ultrasound for killing cancerous cells in the liver or pancreas is to fully destroy the tumor while at the same time widely sparing healthy tissue such as the ribs, colon, stomach, nerves, etc. This requires optimizing transducer parameters such as phase values and pressure amplitude for each single transducer elements to deliver the required energy to the target area in a feasible manner. In particular, it has to be ensured that heating of risk structures like ribs, nerves, gall bladder, intestine etc. is not too high while still delivering enough power to the focal spot. These requirements finally lead to a constrained multi-criteria optimization problem.

## Methods

To solve the constrained multi-criteria optimization problem, the requirements to kill the tumor area and save risk structures have been formulated as objective functions. For example, the destruction of the target tissue can be estimated by the integral of squared pressure amplitude (which is proportional to the heat source) inside the target area. Furthermore, to regularize the problem and to account for the limited power available for the transducer, a penalization term of squared amplitudes is subtracted from the objective function. The Rayleigh-Sommerfeld method is used to compute the pressure field based on the parameters for each transducer element. Finally, the minimization of the objective function leads to an iterative optimization of the transducer parameters such as phase values and amplitudes. The MeVisLab application framework was used to implement the optimization approach.

## Results and conclusions

Initially, the constrained optimization was evaluated on artificial geometric target structures. Gradually, additional artificial risk structures were introduced and finally real data has been used to evaluate the multi-criteria optimization technique. Positive results have been obtained during those simulations. We believe that it will be promising to use our approach for planning of Focused Ultrasound treatments in the future. Additionally, we hope that the multi-criteria optimization technique will help to perform Focused Ultrasound treatments more effectively and efficiently as well as to make such treatments safer for the patients.

